# Thyroid disease diagnosed by point-of-care ultrasound after the induction of anesthesia: a case report

**DOI:** 10.1186/s40981-018-0164-3

**Published:** 2018-03-14

**Authors:** Masafumi Idei, Itaru Watanabe

**Affiliations:** Yokohama Minami Kyosai Hospital, 1-21-1 Mutsuurahigashi, Kanazawa-ku, Yokohama, 2360037 Japan

**Keywords:** Point-of-care ultrasound (POCUS), Perioperative, Thyroid

## To the editor

The importance of point-of-care ultrasound (POCUS) is increasingly recognized [1]. However, although POCUS has been used for perioperative evaluation of the heart, lungs, and airways [1–3], there are few reports on POCUS for perioperative evaluation of the thyroid. We report a case in which POCUS performed by an anesthesiologist in the operating room detected undiagnosed thyroid disease.

## Case presentation

A 57-year-old woman was scheduled for spine surgery. Her preoperative examination was unremarkable, and there were no abnormal findings on blood tests, chest X-ray, and 12-lead electrocardiogram.

**Fig. 1:**
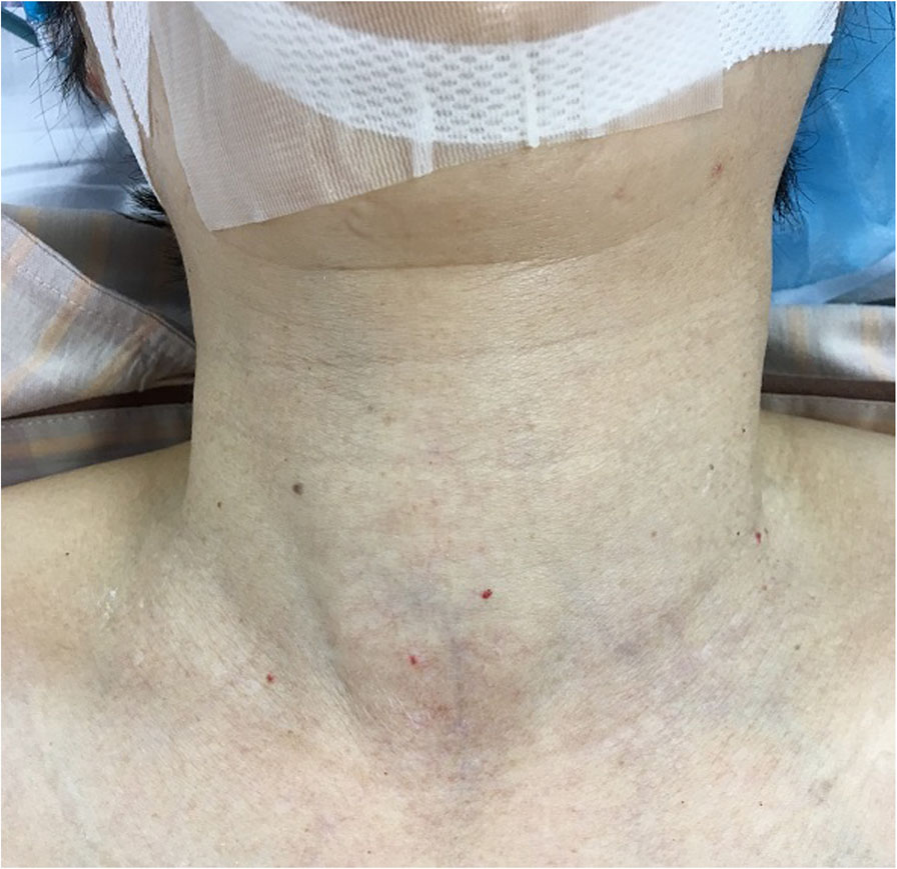
Image shows slight left neck enlargement

**Fig. 2:**
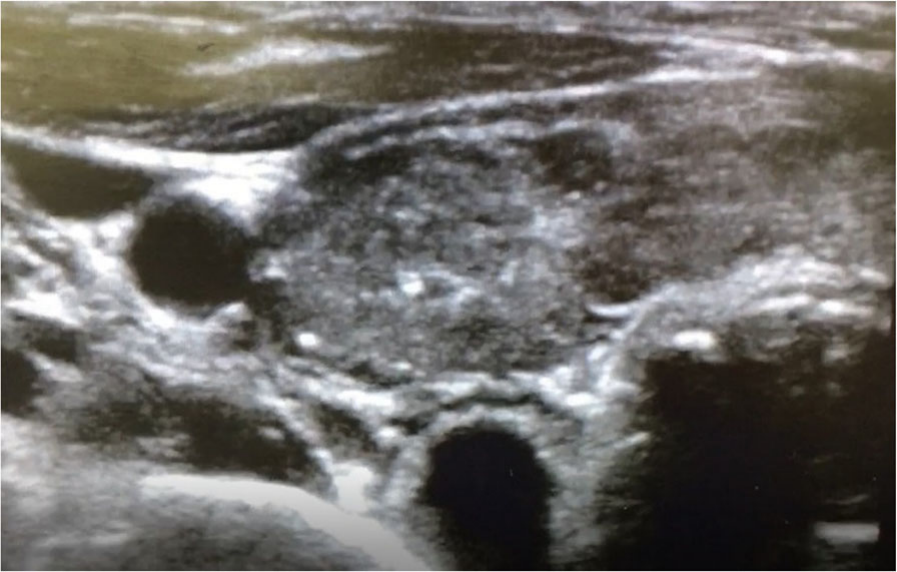
Ultrasonography of the thyroid. The left lobe is heterogeneously enlarged with hypoechoic areas and calcifications

**Fig. 3:**
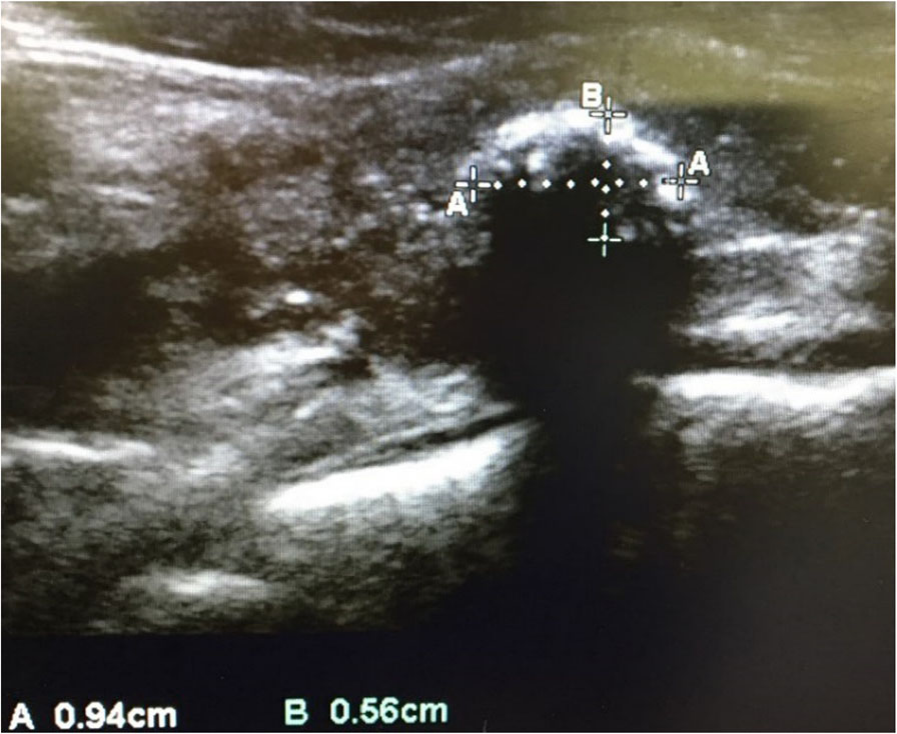
Ultrasonography of the thyroid. A mass with a calcification and an acoustic shadow is observed in the thyroid

Induction of anesthesia was performed with 200 μg of fentanyl, 80 mg of propofol, and 40 mg of rocuronium. Oxygen, air, desflurane, and remifentanil were used to maintain anesthesia. While moving the patient to a prone position, we noticed that her left neck was slightly enlarged (Fig. 1). Using an ultrasound, we found that the left lobe of her thyroid gland was enlarged heterogeneously, with hypoechoic areas and microcalcifications (Fig. 2). The margin of the left lobe was somewhat irregular, and a mass approximately 1 cm in diameter with a calcification and an acoustic shadow was detected (Fig. 3).

An otolaryngologist was immediately consulted, and suspected chronic thyroiditis and papillary adenocarcinoma of the thyroid, and recommended a detailed postoperative examination. Thyroid hormone levels were not measured preoperatively.

Because the patient had no symptoms suggestive of hyperthyroidism or hypothyroidism, no abnormalities on preoperative 12-lead electrocardiogram, and an uneventful course of anesthesia up to that point, we determined that the ultrasound findings would not affect her surgery and decided to continue as scheduled. Total operative time was 143 min, and total anesthesia time was 210 min. She was transferred to the ward after extubation, and the perioperative and postoperative course was unremarkable.

Six days after surgery, the patient underwent otolaryngologic examination and thyroid-specific laboratory testing. The anti-thyroglobulin antibody was high at 562 IU/mL (normal values 0–28 IU/mL) and the anti-thyroid peroxidase antibody was also high, at 40 IU/mL (normal values 0–16 IU/mL). Her thyroid hormones (free triiodothyronine, free thyroxine, and thyroid-stimulating hormone) were within the normal range. Aspiration biopsy cytology revealed chronic thyroiditis (Hashimoto’s disease) and papillary thyroid adenocarcinoma. The left lobe of the thyroid was resected for thyroid carcinoma 7 months after the first surgery, and the patient is currently being followed up in the otolaryngology department.

## Discussion

Routine preoperative measurement of thyroid hormones and imaging of the thyroid is not currently the standard of care, and many patients may undergo surgery with undiagnosed thyroid disease, particularly in emergent cases.

Ultrasonography is commonly used in the diagnosis of thyroid disease [4]. In chronic thyroiditis (Hashimoto’s disease), the gland is enlarged with heterogeneous hypoechoic areas, and the margin of the thyroid gland is irregular [5]. Furthermore, as Hashimoto’s disease progresses, the thyroid gland atrophies and the echo level are attenuated [4].

In Basedow’s disease, the thyroid gland is also enlarged, but the internal echo presents various images from hypoechoic to hyperechoic regions [4]. Another characteristic feature is enhanced blood flow of the suprathyroid artery, as seen on color Doppler [6].

For malignant tumors, such as papillary carcinoma, the outline of the entire thyroid gland is irregular and the internal tissue is heterogeneous [4]. Characteristic findings may include hyperechoic areas with minute calcifications, coarse acoustic shadows, and cervical lymph node metastases [4, 7].

## Conclusions

We present a case in which an anesthesiologist detected thyroid disease using perioperative POCUS. Because thyroid disease can influence perioperative care, we suggest that anesthesiologists should become proficient in the evaluation and diagnosis of the thyroid gland using POCUS.

## Abbreviations

POCUS: Point-of-care ultrasound
